# Hierarchical Processing of Auditory Objects in Humans

**DOI:** 10.1371/journal.pcbi.0030100

**Published:** 2007-06-01

**Authors:** Sukhbinder Kumar, Klaas E Stephan, Jason D Warren, Karl J Friston, Timothy D Griffiths

**Affiliations:** 1 Auditory Group, Medical School, University of Newcastle, Newcastle upon Tyne, United Kingdom; 2 Wellcome Trust Centre for Imaging Neuroscience, Institute of Neurology, University College London, London, United Kingdom; 3 Dementia Research Centre, Institute of Neurology, University College London, London, United Kingdom; University of California San Diego, United States of America

## Abstract

This work examines the computational architecture used by the brain during the analysis of the spectral envelope of sounds, an important acoustic feature for defining auditory objects. Dynamic causal modelling and Bayesian model selection were used to evaluate a family of 16 network models explaining functional magnetic resonance imaging responses in the right temporal lobe during spectral envelope analysis. The models encode different hypotheses about the effective connectivity between Heschl's Gyrus (HG), containing the primary auditory cortex, planum temporale (PT), and superior temporal sulcus (STS), and the modulation of that coupling during spectral envelope analysis. In particular, we aimed to determine whether information processing during spectral envelope analysis takes place in a serial or parallel fashion. The analysis provides strong support for a serial architecture with connections from HG to PT and from PT to STS and an increase of the HG to PT connection during spectral envelope analysis. The work supports a computational model of auditory object processing, based on the abstraction of spectro-temporal “templates” in the PT before further analysis of the abstracted form in anterior temporal lobe areas.

## Author Summary

The past decade has seen a phenomenal rise in applications of functional magnetic resonance imaging for both research and clinical applications. Most of the applications, however, concentrate on finding the regions of the brain that mediate the processing of a cognitive/motor task without determining the interaction between the identified regions. It is, however, the interactions between the different regions that accomplish a given task. In this study, we have examined the interactions between three regions—Heshl's gyrus (HG), planum temporale (PT), and superior temporal sulcus (STS)—that have been implicated in processing the spectral envelope of sounds. The spectral envelope is one of the dimensions of timbre that determine the identity of two sounds that have the same pitch, duration, and intensity. The interaction between the regions is examined using a system-based mathematical modelling technique called dynamic causal modelling (DCM). It is found that flow of information is serial, with HG sending information to PT and then to STS with the connectivity between HG to PT being effectively increased by the extraction of spectral envelope. The study provides evidence for an earlier hypothesis that PT is a computational hub.

## Introduction

**Figure 1:**
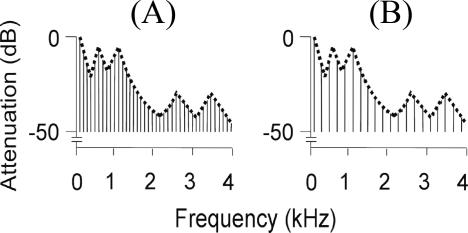
Schematic Frequency Domain Representation of Vowel /a/ at Fundamental Frequency (A) 100 Hz. (B) 200 Hz. In both cases, the same spectral envelope is applied to two different harmonic series patterns.

The concept of an auditory object is controversial [1]. The term can be applied to a sound source like a voice, or an acoustic event generated by a source such as a vowel sound. In both cases, there are features of the object that are independent of the detailed structure of the sound: we can recognise the same vowel, or voice, regardless of the pitch. In these examples, the spectral envelope of the sound determines the particular vowel sound produced, and is, in general, one of the important acoustic features that determine its perceived timbre (Figure 1; spectrogram of the same vowel at different pitch). In this experiment we consider the “abstraction” of the spectral envelope a critical aspect of auditory cognition that defines auditory objects before semantic processing. Such analysis allows generalisation between different exemplars (e.g., the same vowel at a different pitch) in an analogous manner to the generalisation between visual objects that are seen from different perspectives.

The analysis of spectrum in the central auditory system begins in the cochlear nucleus [2], in which models specify sharpening of spectral representation by lateral inhibition [3]. Although relatively less is known about the representation of spectrum in the inferior colliculus and auditory thalamus, a general understanding is that the sharpening of spectrum representation continues in these centres [4]. At the level of the primary cortex, however, animal studies show that representation of spectrum is more complex. Specifically, a given spectrum at the cortex is represented at multiple scales in which a given spectrum has multiple representations at different levels of spectral resolution [5]. Mathematically, this representation has been called ripple analysis, where a given spectrum is decomposed into a sum of ripples of different ripple densities and velocities [6]. Neurons in the primary auditory cortex allow spectral analysis by selectively responding to a fixed ripple density and ripple velocity. The complex spectral analysis in the cortex [7] has not been demonstrated in subcortical areas to the same extent [8]. In this study, we examine the human cortical representation of the spectral envelope independently of the fine structure of the spectrum, a process for which there are a priori grounds for specifying cortical models that are based on initial complex representations in the primary auditory cortex.

We have previously demonstrated bilateral activation of the planum temporale (PT) and right-lateralised activation of the superior temporal sulcus (STS) during the analysis of the spectral envelope in a conventional analysis of functional magnetic resonance imaging (fMRI) data [9]. Such analyses identify the regional nodes of a network that are active during the task without demonstrating the pattern of connections that determines the dynamics of the system: there are multiple mechanisms by which the measured task-induced regional responses could be explained. In the current study, we go beyond classical structure–function correlations and characterise formally the functional interactions between auditory areas involved in spectral envelope analysis. This system identification approach rests on the mathematical characterization of the causal and context-dependent influences that system elements exert upon each other (i.e., effective connectivity [10–13]).

We use dynamic causal modelling (DCM) and Bayesian model selection [14] to address two fundamental questions about the biological computations that attend auditory processing. First, we assess the general structure of the HG–PT–STS network for auditory object processing. In particular, we address the critical question of whether analysis in PT and STS occurs in a serial (hierarchical) fashion, based on connections from HG to PT and from PT to STS, or whether the analysis is based on parallel processing that is mediated by connections from HG to both PT and STS. Second, we address how connection strengths between elements of this cortical network are modulated or enabled during the spectral envelope processing. The approach allows a direct test of a computational mechanism we have suggested previously [15]. This scheme is based on an initial stage of abstraction of the properties of the stimulus that occurs at the PT, before further processing of the abstracted “template” in areas that are concerned with categorical and semantic processing of auditory stimuli. The demonstration of a serial mechanism based on the PT as an intermediate stage would be consistent with such a scheme.

In brief, our results provide strong support for a serial model with increase of the connection strength of the first stage from the HG to PT during spectral envelope analysis. The results suggest a single “stream” for auditory object analysis, and are congruent with macaque models based on a predominant pattern of connectivity from core to belt to parabelt areas.

## Results

**Figure 2:**
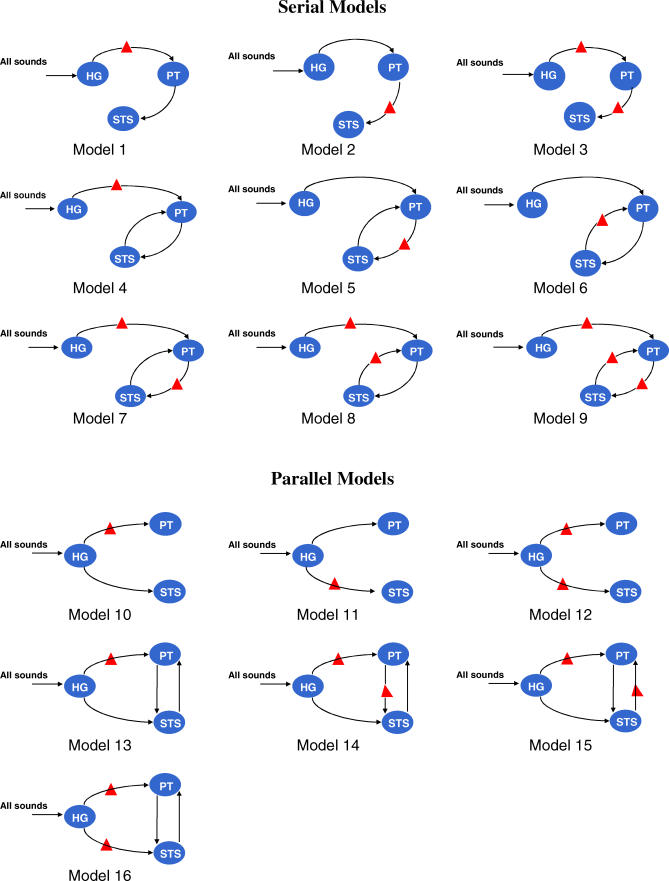
Schematic Representation of Right-Hemisphere Serial and Parallel Models Tested Using DCM The symbol in the pathway between two regions indicates the modulatory effect of extraction of the spectral envelope.

We assessed the ability of different network models to explain the variation in measured fMRI blood oxygenation level–dependent (BOLD) responses in the HG, PT, and STS in the right hemisphere during the extraction of the spectral envelope of generic sounds without any semantic association. Two broad classes of models, serial and parallel, were defined as shown in Figure 2. The serial models contain connections from HG → PT and thence from PT → STS. In contrast, parallel models postulate connections from the HG to both the PT and STS. The models within each family differ with respect to the back connections specified, and with respect to the specific site of the modulatory effect of spectral envelope analysis. The models were compared using Bayesian model selection implemented within SPM (http://www.fil.ion.ucl.ac.uk/spm/software/spm5). The selection procedure estimates the probability of each model given the data using Akaike information criterion (AIC) and Bayesian information criterion (BIC) approximations to each model's log-evidence or marginal likelihood.

**Figure 3:**
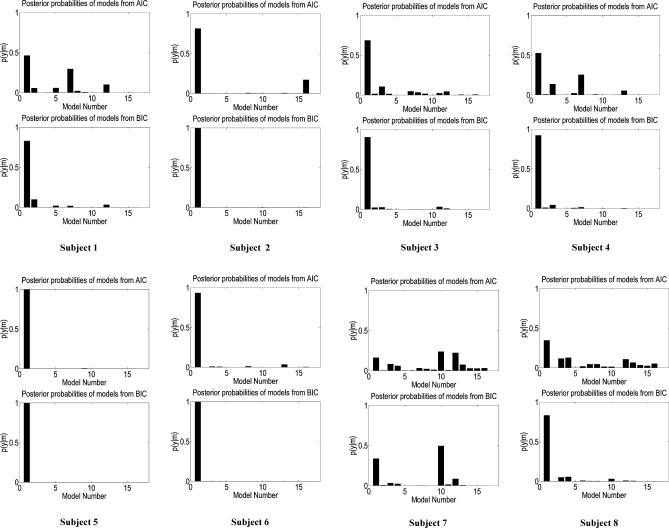
Plots of Probabilities *p*(*m* | *y*) for Individual Participants for the 16 Models Included in DCM The probabilities have been normalised so that they sum to one. These represent the probability of the model, given the data, assuming each model is, a priori, equally likely.

Figure 3 shows the evidence for the models, determined separately using AIC and BIC, in eight participants. In this figure, we have assumed all models were equally likely a priori. This allows us to treat the normalised marginal likelihood as the conditional probability of each model. Model 1 is the optimal model over all participants, with the exception of participant 7. The parameters for this model specify a serial model with connectivity (HG → PT → STS) and modulation of connection from HG → PT during the analysis of the spectral envelope. In addition to the individual inference, Table 1 shows the group Bayes factor (GBF) for model 1 with respect to the other 15 models. Given candidate hypotheses (models) *i* and *j*, a Bayes factor of 150 corresponds to a belief of 99% in the statement that “hypothesis *i* is true”. Following the usual conventions in Bayesian statistics [14,16], this corresponds to “strong” evidence in favor of model *i* (compare Table 2). All the values of the GBF for model 1 with respect to all other models is greater than 150, corresponding to very strong evidence for the serial model number 1. Plots of measured and predicted BOLD time series for a single participant are shown in Figure S3. This figure shows that the BOLD response in all three areas, particularly in STS, is fitted well by the optimal model. This demonstrates that (1) activity in the PT can be explained as a function of the input from the HG and its modulation during spectral envelope processing, and that (2) STS activity can be explained as a function of the input from the PT (compare the structure of model 1 as shown in Figure 2).

**Table 1:**
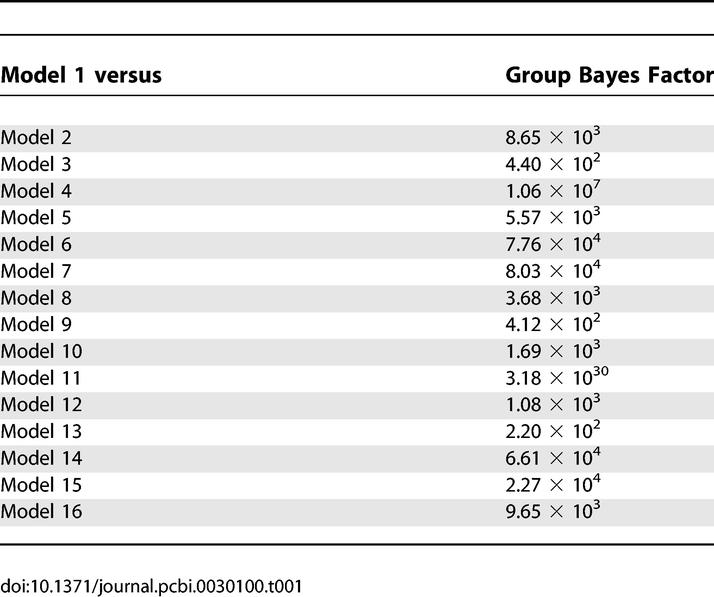
Group Bayes Factor for the Optimal Model (the Serial Model 1)

**Table 2:**
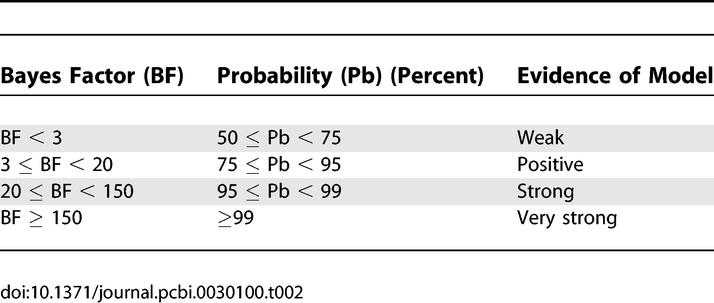
Relation between Bayes Factor and Evidence of Model

Estimates of the interregional connection strengths and their modulation for each participant and probabilities that the coupling estimates are greater than zero are shown in Tables 3 and 4, respectively. The probabilities that the connection strengths are greater than zero are all ~1.00, with the exception of the PT → STS connection in participant 5. Furthermore, the probability that the modulation of the strength of the connection from HG → PT is greater than zero is ~1.00 in all participants except 1 and 7, where the probability is greater than 0.9. A further *t*-test was carried out on intrinsic and modulatory connection strengths to assess the group level connection strengths. The mean values of HG → PT and PT → STS intrinsic connection strengths are 0.37 (*p* < 0.01) and 0.48 (*p* < 0.01), respectively. The mean value of modulatory HG → PT (measured in percentage increase) in connection strength is 109.29 (*p* < 0.01)

**Table 3:**
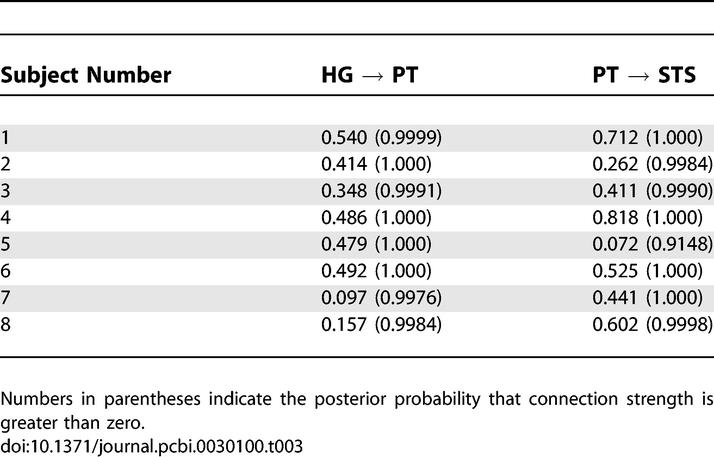
Intrinsic Connection Strengths for the Optimal Model

**Table 4:**
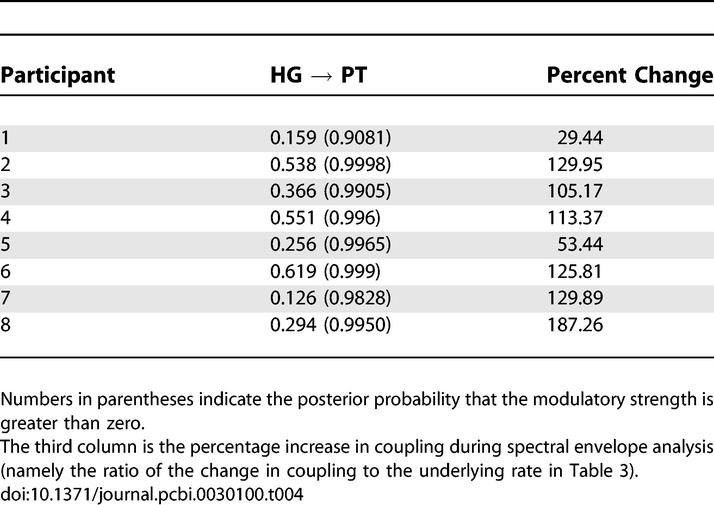
Strength of the Modulation of the HG → PT Connection by Spectral Envelope Processing

Theoretically, a large number of models other than those considered above are possible. The choice of these models was motivated by preliminary analysis of the data. This analysis showed that (1) inclusion of modulation of the HG → PT pathway is critical to model performance (as evaluated using AIC and BIC), and (2) addition of a feedback path (from PT → HG) led to poorer model performance. Since AIC and BIC strike a tradeoff between predictability and cost (measured in terms of number of parameters) of the model, this implies that the feedback path does not significantly increase the predictability but adds to the cost. We have estimated 54 further models that include back connections to HG from PT and STS (in both serial and parallel models) and HG → STS → PT models. These models, which are anatomically and functionally plausible, are schematically represented in Figure S1. A plot of posterior probabilities of all the 70 models, with the first 16 as shown in Figure 1 and the next 54 as shown in Figure S1, is shown in Figure S2.

On evaluating all the 70 estimated models (Figure S2), participants 1 to 6 continued to show very strong evidence in favour of model 1. In participants 7 and 8, however, the model selection procedure failed to identify an optimal model, although for different reasons. In participant 7, there was no decisive evidence in favour of any model: the Bayes factor for comparing model 10 to model 1 (the latter being the optimum model in the first six participants) was only 1.4. This designates very little evidence in favour of model 10 and is substantially below the threshold (i.e., 3) that is commonly used in Bayesian statistics to decide between two models [16]. In contrast, in participant 8, the two approximations to the model evidence (AIC and BIC) favoured different models (21 and 19, respectively). Similarly, model 1 was superior to model 21 according to the BIC criterion, but inferior according to the AIC criterion. These contradictory constellations represent a limitation of the model selection procedure adopted here, which, in cases like this particular participant, prevents one from drawing a firm conclusion about which model is optimal [14]. Overall, therefore, six out of eight participants showed strong evidence in favour of model 1, and the remaining two participants failed to show consistent evidence in favour of any one model.

## Discussion

A major challenge in auditory cognition is to relate cognitive processes to dynamic interactions among cortical regions. DCM was designed specifically for functional imaging data to model and draw inferences about effective connectivity between different regions. The present study aimed to understand the systems-level organisation of the computational mechanisms in the HG, PT, and STS invoked for the analysis of the spectral envelope of sounds. Two broad categories of models, serial and parallel, were specified a priori. The data provide very strong evidence for a serial model in which analysis of the spectral envelope specifically enhances the connection from the HG to the PT.

In contrast to the visual system [17–22], the effective connectivity between auditory areas has not been studied extensively. The few previous studies used structural equation modelling (SEM; [23–25]). The present study is the first to use DCM to examine the auditory system. Effective connectivity between the HG and the PT was suggested by a previous study [23] using SEM. However, the results were inconsistent across participants and also between the group analysis and individual participant analyses. Another study using SEM [25] considered the connection between the HG and PT and frontal areas, but did not examine local connection with the STS. In the present study, we have provided evidence for a consistent model across participants based on serial analysis in the HG, PT, and STS. The group analysis also concurs with the individual participant analysis. We now consider the limitations of the model supported by our data, and its biological significance.

DCM models the causal influence of the neural activity in one area on another, where those areas have a direct or indirect anatomical connection. The connections that we have specified a priori are plausible, given available macaque data [26], but data on the interconnections between human cortical areas are limited to a small number of postmortem dye-tracing studies [27] and “opportunistic” studies of neurosurgical patients [28]. The model supported here is both consistent with the existence of direct anatomical projections between the HG and the PT and between the PT and the STS, and also provides evidence for the functional expression of these projections. The connection between the HG and the PT is supported by the neurophysiological evidence and tracing studies above, but further evidence for the anatomical connection between the PT and the STS, particularly, is required. Such evidence might accrue from further postmortem work or the application of in vivo techniques such as diffusion tensor imaging.

In this study, we have tested the simplest possible model to describe the data in individual participants. In particular, the HG volume we have used is likely to contain primary and secondary areas: we have previously argued [29] that there are three functional areas in the HG that correspond to the three macaque “core” areas A1, R, and RT. Kaas and Hacket [26] have described a macaque scheme based on a pattern of connectivity that extends from core to belt to parabelt areas. The connectivity structure of the serial model that was selected as optimal in this study is consistent with such a scheme, if PT contains the homologues of belt areas. However, the detailed pattern of interconnections within the HG could not be assessed in this dataset, as three distinct functional areas in the HG were not demonstrated in all the individual participants. The extent to which the analysis may involve several different functional areas within the HG before analysis occurring in the PT therefore cannot be determined.

Like the HG, the PT is a large anatomical area, corresponding to the cytoarchitectonic area Te 3.0 [30], within which there may be a number of functional subdivisions. Homology with the macaque becomes even more difficult than in the case of core areas. One possibility is that there may be “belt homologue” areas in the PT adjacent to the three “core” areas suggested in the HG. The connectivity between the HG and the PT identified in this analysis would then be broadly congruent with the core-to-belt projections that have been identified in the macaque [26]. Recording work in the macaque suggests that more anterior belt areas are critical for auditory object analysis, although the distinction between anterior and posterior auditory areas is not as marked as in the case of spatial analysis [31]. Connections to the STS are much more difficult to characterise in terms of homology, especially in view of the existence of three temporal gyri in the human and two in the macaque. Human functional data for the STS demonstrate complex cognitive analysis, including voice processing [32] and the integration of auditory and visual object information [33]. Whether or not the macaque homology holds, however, the other human studies suggest a role for the STS in associative analysis, whereas the serial analysis we have demonstrated is also hierarchal: perceptual analysis in the earlier areas (HG and PT) precedes more complex associative analysis in the later area (STS).

The model identified as optimal by our Bayesian selection procedure is characterised by a serial architecture in which high activity in higher auditory areas during spectral envelope extraction is explained by a modulation of the HG → PT connection. In neurophysiological terms, this means that only the HG → PT connection is dependent on the spectral envelope modulation, and that the induced context-dependent response in the PT is simply relayed on to the STS. In functional terms, this means that spectral envelope analysis is likely to be completed at the stage of the PT, and that the differential responses in the STS are a downstream reflection of this process. In contrast, if we imagine that model 6 (compare Figure 2) had been selected as optimal, the interpretation would have been that context-dependent connectivity was restricted to the STS → PT connection and that functionally, spectral envelope analysis is likely to be performed at the level of the STS and the results fed back to the PT via the STS → PT connection.

Completion of spectral envelope analysis at the PT in the absence of a task is consistent with the “obligatory” abstraction of templates before the PT that does not depend on the existence of a task. It will be of interest in future studies to see if the presence of an active task produces modulation of the second serial stage between the PT and the STS. This is also interesting in terms of the idea that the PT may be a critical computational “hub” where spectro-temporal “templates” are extracted before analysis in higher centres that assess the significance of a particular template (such as its relevance to position in space or semantic category) [15]. This abstraction is homologous to feature extraction or selection in machine learning: feature selection is a process commonly used in machine learning, in which features available from the data are selected for subsequent inference and learning. The spectral envelope corresponds to a type of template that is independent of the spectral fine structure of the sound, and is important for source identification independent of the pitch of the source or whether it was producing a harmonic sound or noise.

There are certain limitations of the models that have been tested in the present work. First, only cortical connections have been considered. The thalamic connections were not included in the models, because of (1) the absence of activation in the auditory thalamus due to our experimental manipulation and (2) the evidence from animal work that complex spectral analysis first occurs in the primary auditory cortex [8]. Also, only models of the right hemisphere have been considered here, and hemispheric interactions have been ignored. This is because the conventional fMRI analysis of spectral envelope processing has consistently demonstrated a dominant role of the right hemisphere, with substantially less involvement of the left hemisphere.

## Materials and Methods

### Stimuli.

**Figure 4:**
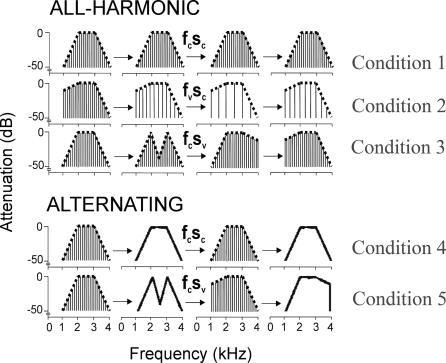
Schematic Representation of Stimuli Used in the fMRI Experiment Sequences are either “all-harmonic”, in which the elements of sequence are harmonic sounds, or “alternating”, in which the elements alternate between harmonic and noise stimuli. In condition 1, the spectral envelope and pitch (fundamental frequency) are fixed; in condition 2, the spectral envelope is fixed, but pitch is varying; in condition 3, the spectral envelope is varying, but pitch is fixed. Conditions 4 and 5 are “alternating” sequences. In condition 4, harmonic and noise sounds alternate with fixed spectral envelope; in condition 5, harmonic and noise sounds alternate with varying spectral envelope. f_c_S_c_, f_0_ constant, spectral envelope constant; f_v_S_c_, f_0_ varying, spectral envelope constant; f_c_S_v_, f_0_ constant, spectral envelope varying.

Sequences of harmonic or noise stimuli were synthesised digitally at a sampling frequency of 44.1 kHz and 16-bit resolution. The harmonic stimuli were harmonic series, whereas the noise stimuli were random-phase noise. The stimuli were synthesised in the frequency domain, allowing the same spectral envelope to be applied to either harmonic or noise sounds. The duration of stimuli was 500 ms (with a 20-ms gating window). Synthesized sounds were used to form two sets of sequences. The first set, called “all-harmonic”, consisted of harmonic sounds only; the second set, known as “alternating”, consisted of alternating harmonic and noise sounds. The “all-harmonic” set has three experimental conditions: (1) the spectral envelope and pitch (fundamental frequency) of the sounds in the sequence are fixed; (2) the spectral envelope is fixed, but the fundamental frequency of sounds in the sequence is changing; and (3) the spectral envelope is changing, but fundamental frequency is fixed. The fundamental frequencies of the sounds in this set are 120, 144, 168, or 192 Hz, either fixed or varied between successive sounds in the sequence. The “alternating” set has two conditions: (1) harmonic and noise sounds alternating with fixed envelope and (2) harmonic and noise sounds alternating with changing spectral envelope. In total, the experiment has six conditions, five as described above, and the silence condition. The conditions are schematically shown in Figure 4. Change in fundamental frequency f_0_ is perceived as change in pitch, whereas change in the spectral envelope is perceived as a change in the identity of the source. The critical contrast in the group analysis to assess the “extraction” of the spectral envelope is the contrast between the two alternating conditions with changing and fixed spectral envelopes when the fine spectral structure of the stimuli is continually changing*.* The difference between these two conditions corresponds to an alteration in the perceived source over and above the low-level analysis of the fine spectral structure*.* That contrast was used to define spectral envelope extraction at the group level. The total duration of each sequence was 7.5 s or 8 s.

Before carrying out the fMRI experiment, the participant's ability to perceive the change in the spectral envelope was assessed in a separate psychophysical experiment. The same elements of the sequences used in the fMRI experiment were presented to the participants in a two-interval–two-alternative forced-choice paradigm. The task was to detect change in pitch (in all harmonic sequences) or change in the spectral envelope (all-harmonic or alternate conditions). Participants were able to detect harmonic sequences with pitch change or spectral shape change with 100% accuracy. Participants were also able to detect change in the spectral envelope in (alternating condition) with 100% accuracy. The changes in spectral shape therefore could be reliably detected independent of fine spectro-temporal changes.

### fMRI data acquisition and processing.

Data from eight healthy volunteers were used for DCM. All participants gave their informed consent, and the experiment was carried out with approval of the local ethics committee.

fMRI data were acquired from a 1.5-T Siemens SONATA system (http://www.siemens.com) using gradient echo planar imaging (echo time = 50 ms; flip angle = 90 degrees) in a sparse image acquisition protocol [34]. Stimuli were presented diotically at a fixed sound-pressure level of 80 dB during the silent phase of the protocol. A whole-brain volume of 48 slices (2-mm thickness, in plane resolution 3 × 3 mm^2^) was acquired every 12.5 s with a time for acquisition of 4.32 s. Participants were instructed to attend to the stimuli with their eyes closed. In a typical trial of image acquisition, stimulus is first presented for about 8 s, followed by image acquisition that lasts 4.32 s. There was no active auditory discrimination task, however; to maintain attention, participants were asked to signal the end of each sequence by pressing a button box under the right hand. The experiment was divided in two runs, with 16 scans acquired for each condition in each run. The order of conditions was fully randomised.

Images were realigned, normalized to a standard EPI template, and smoothed with a 3-D Gaussian kernel with full-width half-maximum of 8 mm. Regressors for the design matrix were created by convolving boxcar stimulus functions (representing stimulus events) with a canonical hemodynamic response function. Linear contrasts of parameter estimates were created for each participant. Finally, a random-effects group analysis was performed by comparing the participant-specific contrast images with the appropriate *t*-tests to produce a statistical parametric map.

### Selection of volumes of interest.

The general goal of DCM is to provide mechanistic explanations, in terms of connectivity and its modulation, for local effects observed in a conventional univariate analysis. The SPM results of the present data demonstrated a neural system in which the lowest level (i.e., the primary auditory cortex in the HG) does not show any significant activity differences between experimental conditions, but is uniformly driven by auditory stimulation. In contrast, higher auditory areas (PT and STS) show higher activity when spectral envelope extraction is required. These two observations could be potentially explained by a network model (i.e., DCM) in which a “neutral” input area, perturbed by auditory stimuli per se, drives two higher auditory areas differentially (i.e., some or all of the efferent connections of the input area are modulated by spectral envelope extraction).

Our DCM included three areas (HG, PT, and STS) in the right hemisphere. These areas were identified for each participant based on the coordinates of the peak activation obtained in the group analysis. For the HG, the contrast (condition 4 + condition 5) versus silence was used to define the centre of the volume from which the time series was extracted. For the PT and the STS, the contrast between the alternating sequences with variable and fixed spectral envelopes (condition 5 versus condition 4; see Figure 4) was used to define the centres of the volumes. The centre of each volume (defined as a sphere of 4-mm radius) was located at the local maximum that was nearest to the peak coordinates in the group analysis. The selected local maximum was constrained to lie within 16 mm (twice the width of the Gaussian smoothing kernel) of the group peak coordinates and within the same anatomical gyrus/sulcus as the group activation.

The coordinates of peak activation for the three volumes in each participant are given in Table 5. A summary time series from each of the three regions was furnished by the principal eigenvariate of measurements recorded from all significant voxels located within the volume.

**Table 5:**
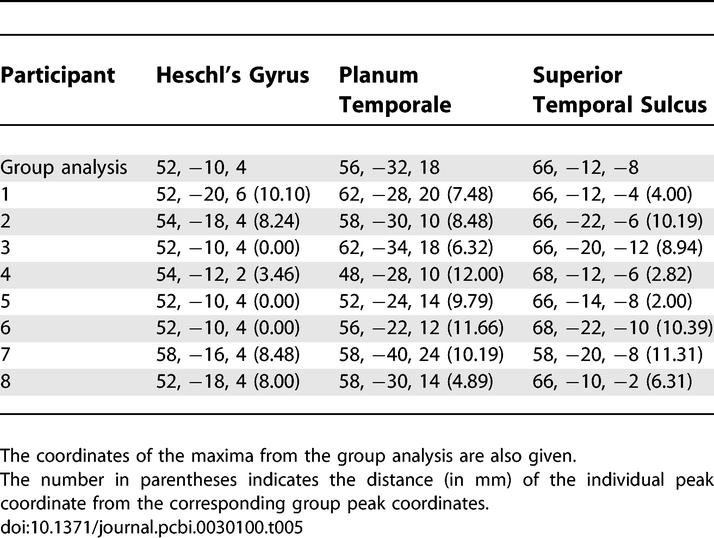
Peak Coordinates for the Three Regions Used in DCM

### Dynamic causal modelling.

From a system theory point of view, the brain can be treated as a nonlinear input–output dynamic system that can be excited by controlled stimuli and which response (hemodynamic response here) can be measured. The central idea behind DCM is to estimate and draw inferences about the causal interaction between different regions of the brain by identifying a model for the system using input–output measurements.

In DCM, three different sets of parameters are used. The first set of parameters, known as intrinsic parameters, models the anatomical or hardwired connection strengths between the regions. These parameters represent the influence that one region has over the other in the absence of any external excitation of the system. The second set of parameters, known as modulatory parameters, models the change in intrinsic connection strength that is induced by the external experimental input. These parameters are therefore input-specific and are also referred to as “bilinear terms or parameters.” The third set of parameters models the direct influence of an external stimulus on a given region. The conventional general linear model analysis is based on the assumption that any external stimulus has a direct influence on a region; therefore, it is the third set of parameters on which a general linear model analysis is based exclusively. DCM, therefore, can also be regarded as more general, with the general linear model analysis being a specific situation in which the interaction parameters (first and second sets) are assumed to be zero.

DCM has several advantages over other models of effective connectivity (e.g., SEM [20], multivariate autoregression [35], or Granger causality [36]; see [13,37] for details). For example, DCM takes temporal order (and autocorrelation of the fMRI time series) into account. It further allows one to model the effects of experimentally controlled manipulations as either affecting regional activity directly (e.g., sensory inputs) or modulating the strengths of connections, and does not need to assume that the system is driven by stochastic innovations. Most important, however, DCM is currently the only model of effective connectivity that combines a neural population model with a biophysical hemodynamic forward model, and is thus able to model how system dynamics at the (hidden) neuronal level translates into measured BOLD signals.

In brief, DCM is based on a bilinear model of neural population dynamics that is combined with a hemodynamic model [38,39], describing the transformation of neural activity into predicted BOLD responses. The neural dynamics are modelled by the following bilinear differential equation:

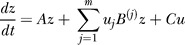
where *z* is the state vector (with one state variable per region), *t* is continuous time, and *u_j_* is the *j*-th experimental input to the modelled system (i.e., some experimentally controlled manipulation). This state equation represents the strength of connections between the modelled regions (the *A* matrix), the modulation of these connections as a function of experimental manipulations (e.g., changes in task; the *B* ^(1)^...*B* ^(*m*)^ matrices), and the strengths of direct inputs to the modelled system (e.g., sensory stimuli; the *C* matrix). These parameters correspond to the rate constants of the modelled neurophysiological processes. Combining the neural and hemodynamic model creates a joint forward model, which is inverted using conventional techniques (expectation maximisation) to give the posterior density of the parameters. Under Gaussian assumptions, this density can be characterised in terms of its maximum a posteriori estimate and its posterior covariance. This density obtains by optimising a free-energy bound on the models log-evidence or marginal likelihood.

### Choice of models.

DCM is a hypothesis-driven technique in which model space is specified a priori. The first objective of the present study was to test if the coupling between the HG, PT, and STS is serial or parallel. To address this, two broad categories of models, serial and parallel, were specified (Figure 2). In the serial models, auditory inputs entering the HG reach the STS via the PT, and, thus, processing in the STS depends on inputs from the PT. In contrast, in the parallel models, the HG connects to both the PT and the STS, thus enabling a parallel processing in the PT and STS. The second objective was to determine where, in the best model, task requirements (i.e., spectral envelope analysis) led to changes (i.e., modulation) in the connection strengths. The modulatory input is defined as condition 5 of the experiment. In total, 16 models (nine serial, seven parallel) were inverted and compared using their log-evidence. These models are shown in Figure 2. To rule out the possibility of other theoretically possible models, 54 additional models shown in Figure S2 were also estimated, and their log-evidence was computed.

### Selection of optimal model.

A general problem that arises in any modelling exercise is to decide, given some data, which of several competing models is optimal. A number of criteria have been proposed in the modelling literature [40]. From a Bayesian perspective, an optimal criterion is the model evidence (i.e., the probability p(*y* | *m*) of obtaining the data *y* given a particular model *m* [16]). Critically, the model evidence not only takes into account the relative fit of competing models, but also their relative complexity (i.e., the number of free parameters). This is important because there is a tradeoff between the fit of a model and its generalizability (i.e., how well it explains different datasets generated from the same underlying process). As the number of free parameters is increased, model fit increases monotonically, whereas beyond a certain point, model generalizability decreases. The reason for this is “overfitting”: an increasingly complex model will, at some point, start to fit noise that is specific to one dataset and thus become less generalisable across multiple realizations of the same underlying generative process.

As the model evidence cannot always be derived analytically, two commonly used approximations are the AIC and the BIC [14]. These approximations, however, do not necessarily give identical results because the BIC favours simpler models, whereas the AIC is biased toward more complex models. Here, we have adopted the usual conventions (compare [14]) (1) that a conclusion can only be drawn if these two criteria agree, and (2) that the more conservative of the two estimates is chosen. Finally, the relative evidence of one model as compared with another is expressed by the so-called “Bayes factor”:

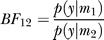
where *BF*_12_ is the Bayes factor of model 1 with respect to model 2.

Following the selection of a best model for each participant, the optimal model for a group of participants can be determined by the GBF, which is the product of the Bayes factors for each individual participant [41].

## Supporting Information

Figure S1 Schematic Representation of Models Estimated To Rule Out the Possibility of Other Theoretical Models Than Those Tested in the Main Text The symbol in the pathway between two regions indicates the modulatory effect of extraction of the spectral envelope.
(14 KB PDF)

Figure S2 Plots of Probabilities *p(m* | *y*) Determined Using AIC and BIC for All 70 Models The first 16 models are as shown in Figure 2, and the next 54 models are as shown here.
(92 KB PDF)

Figure S3 Time Series Plots of Measured and Predicted BOLD Responses for Participant 5 (A) HG. (B) PT. (C) STS. (70 KB DOC)

## Abbreviations

AIC: Akaike information criterion
BIC: Bayesian information criterion
BOLD: blood oxygenation level–dependent
DCM: dynamic causal modelling
fMRI: functional magnetic resonance imaging
GBF: group Bayes factor
HG: Heschl's Gyrus
PT: planum temporale
SEM: structural equation modelling
STS: superior temporal sulcus
