# Supplementary material for: Hierarchical Processing of Auditory Objects in Humans
Source: PLoS Comput Biol. 2007 Jun 1;3(6):e100. doi: 10.1371/journal.pcbi.0030100 (PMC1885275; doi:10.1371/journal.pcbi.0030100)
Supplement: Supplementary file 1 [file pcbi.0030100.sg001.pdf]

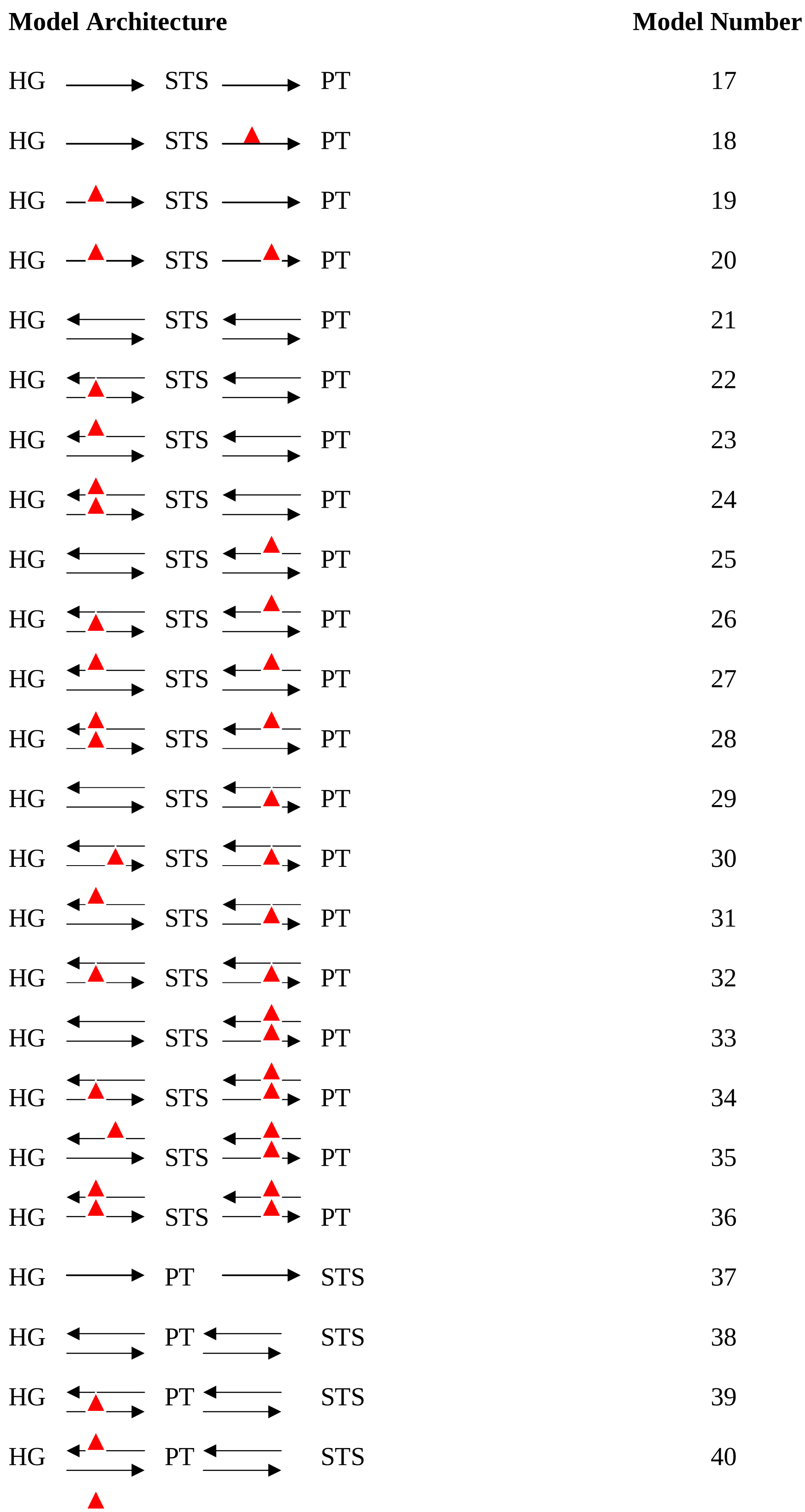
Model Architecture
Model Number
HG STS PT 17
HG STS PT 18
HG STS PT 19
HG STS PT 20
HG STS PT 21
HG STS PT 22
HG STS PT 23
HG STS PT 24
HG STS PT 25
HG STS PT 26
HG STS PT 27
HG STS PT 28
HG STS PT 29
HG STS PT 30
HG STS PT 31
HG STS PT 32
HG STS PT 33
HG STS PT 34
HG STS PT 35
HG STS PT 36
HG PT STS 37
HG PT STS 38
HG PT STS 39
HG PT STS 40

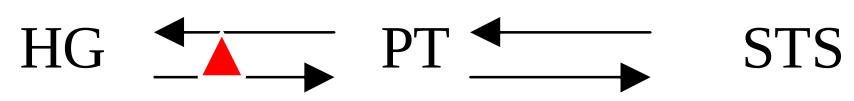

41

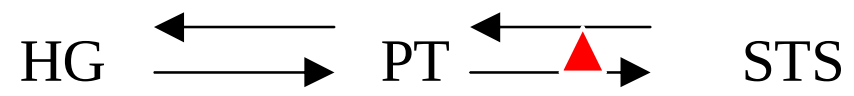

42

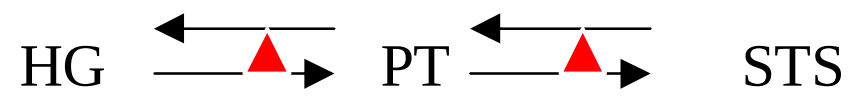

43

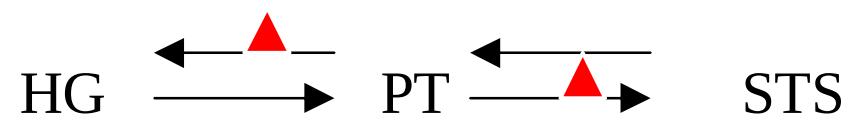

44

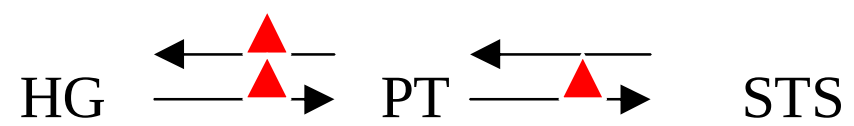

45

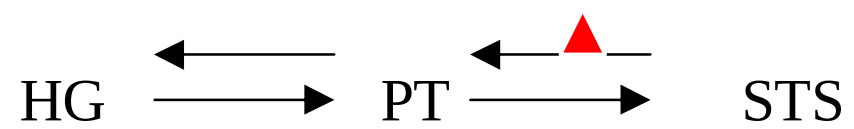

46

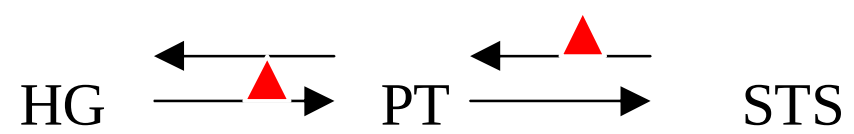

47

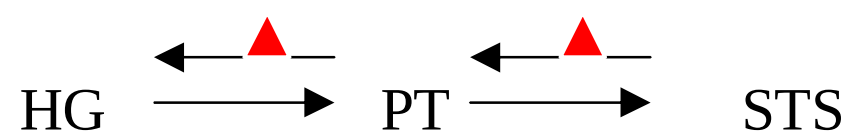

48

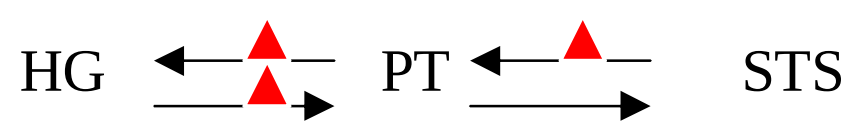

49

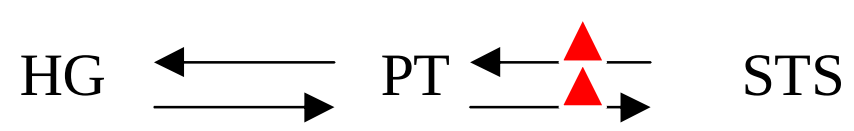

50

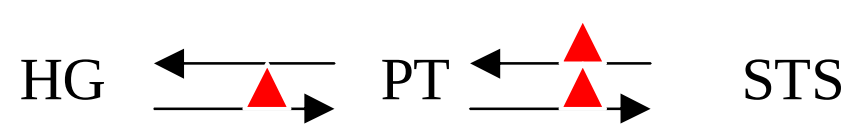

51

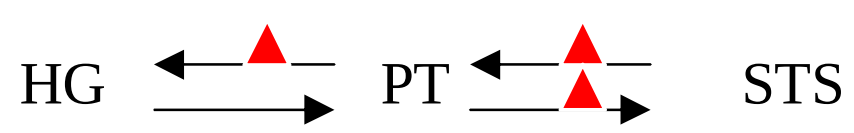

52

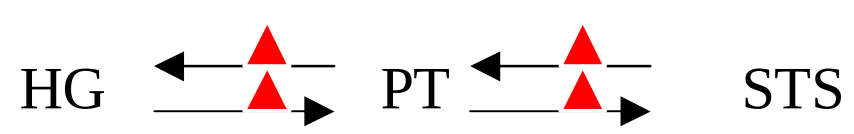

53

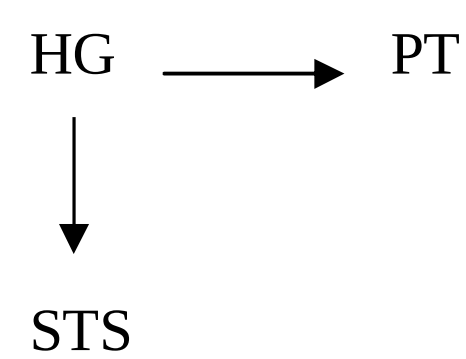

54

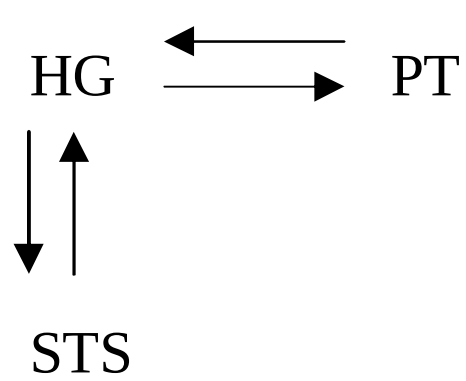

55

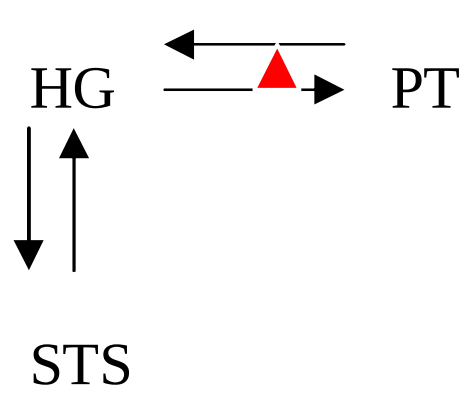

56

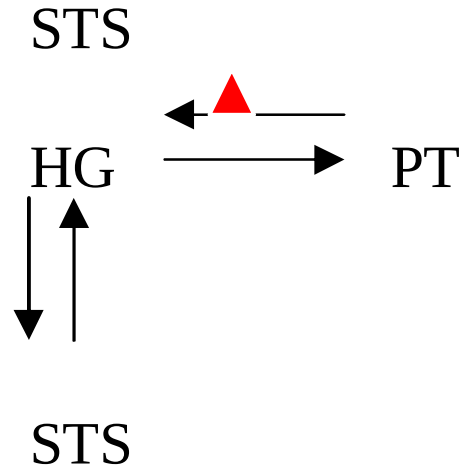

57

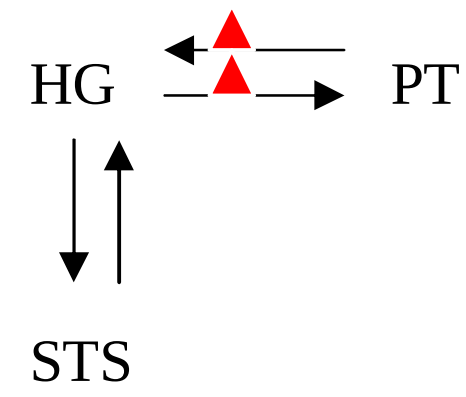

58

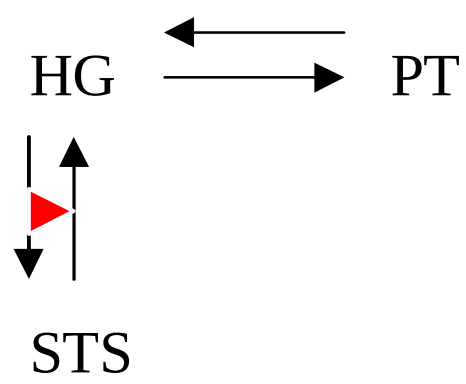

59

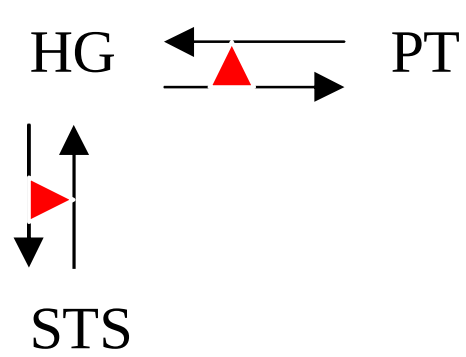

60

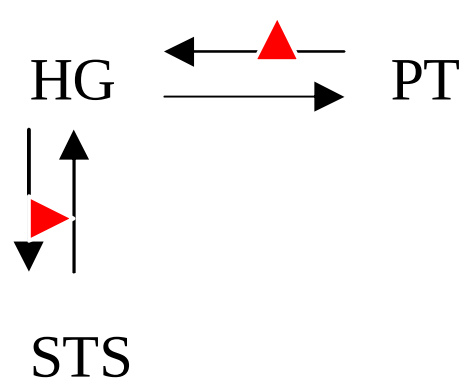

61

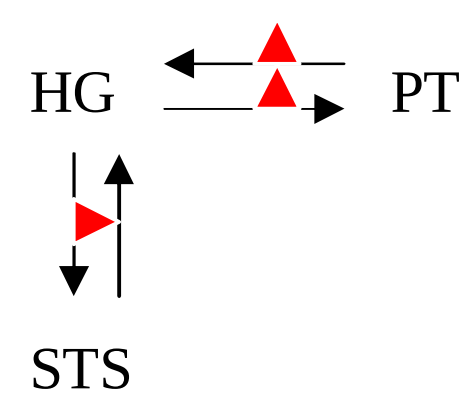

62

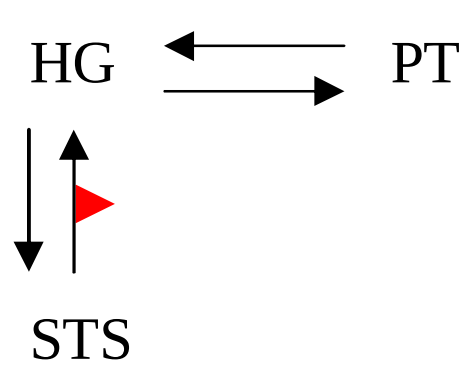

63

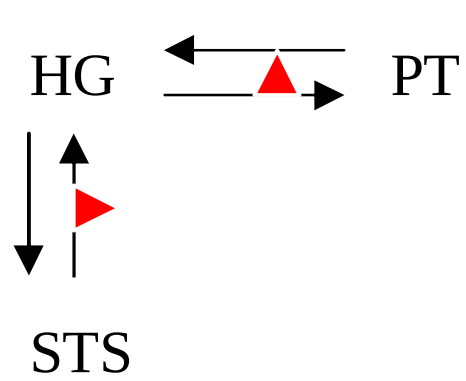

64

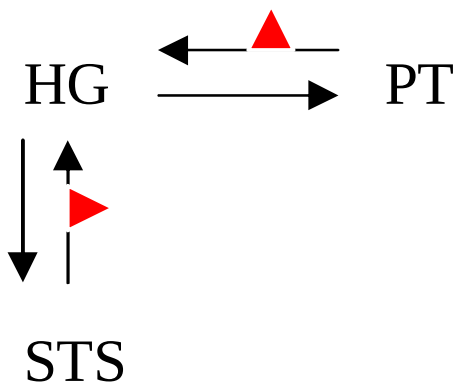

65

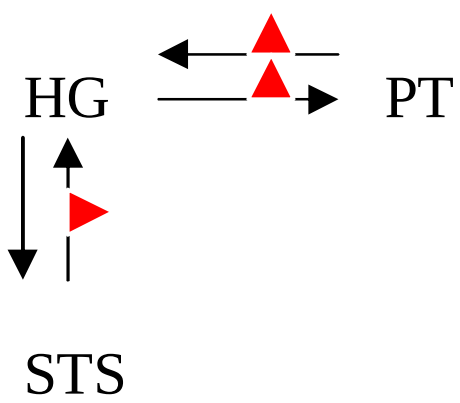

66

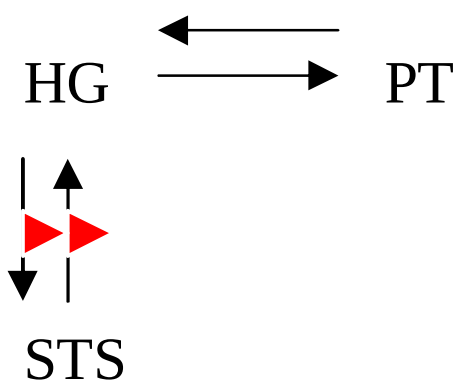

67

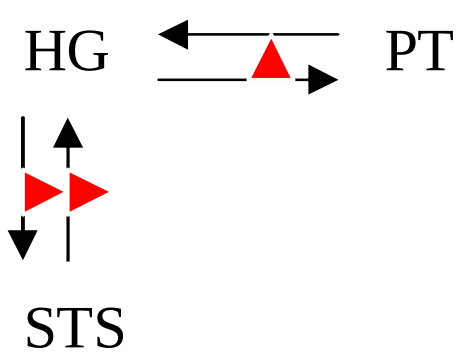

68

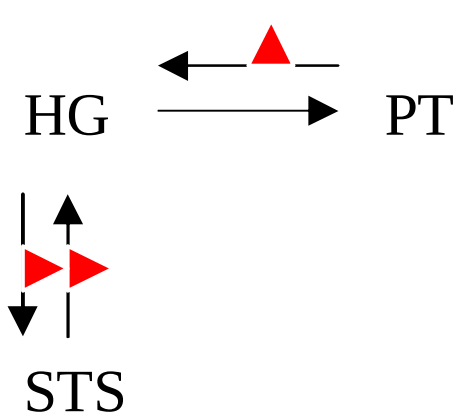

69

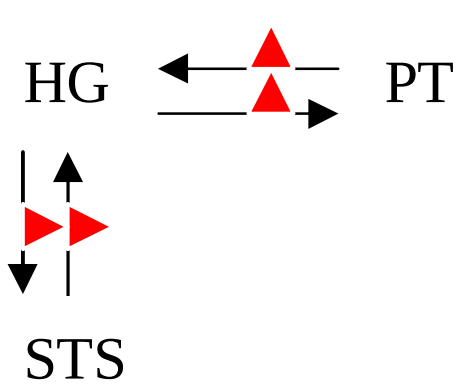

70
