# Supplementary material for: Hierarchical Processing of Auditory Objects in Humans
Source: PLoS Comput Biol. 2007 Jun 1;3(6):e100. doi: 10.1371/journal.pcbi.0030100 (PMC1885275; doi:10.1371/journal.pcbi.0030100)
Supplement: Supplementary file 2 [file pcbi.0030100.sg002.pdf]

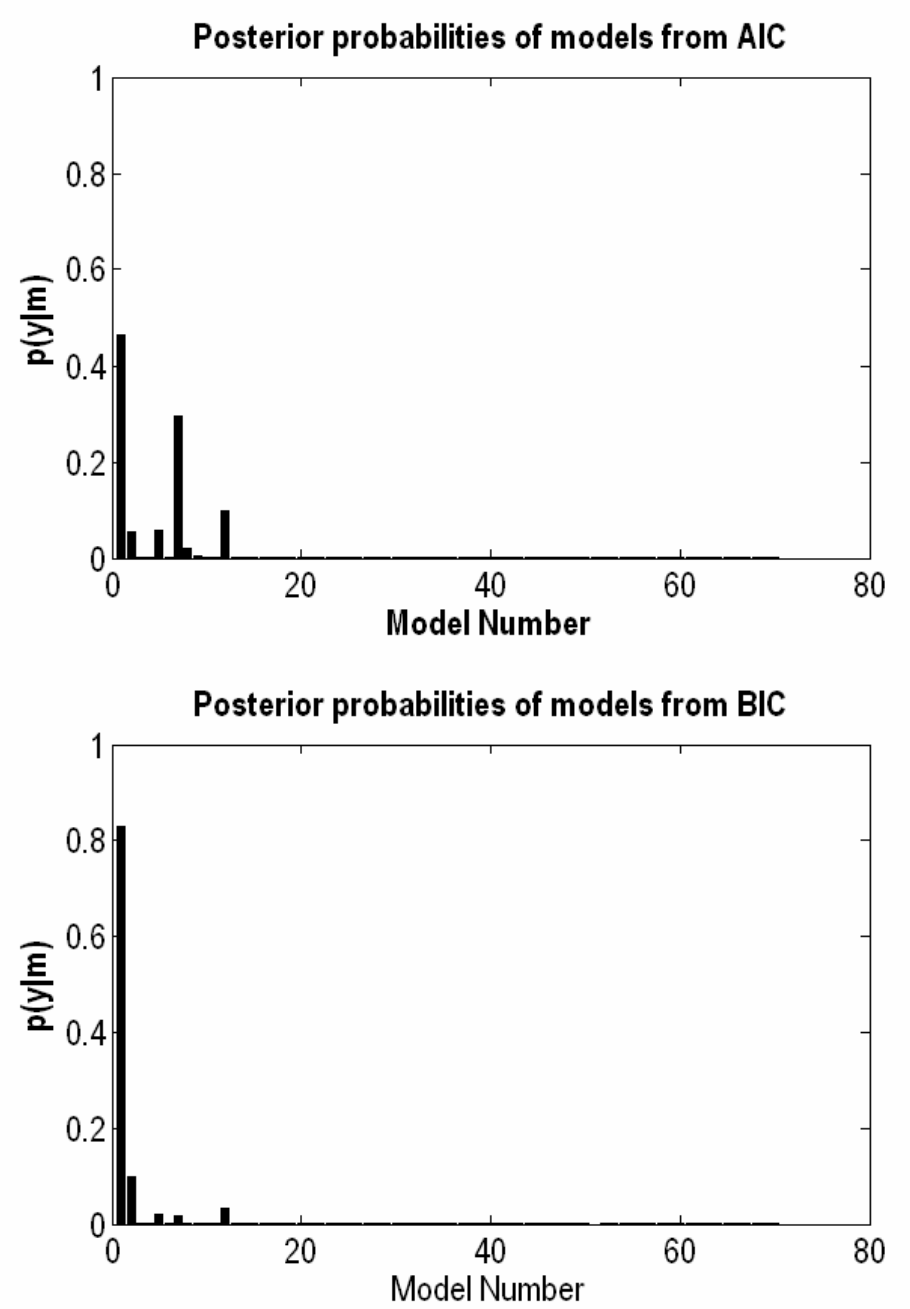

Subject 1

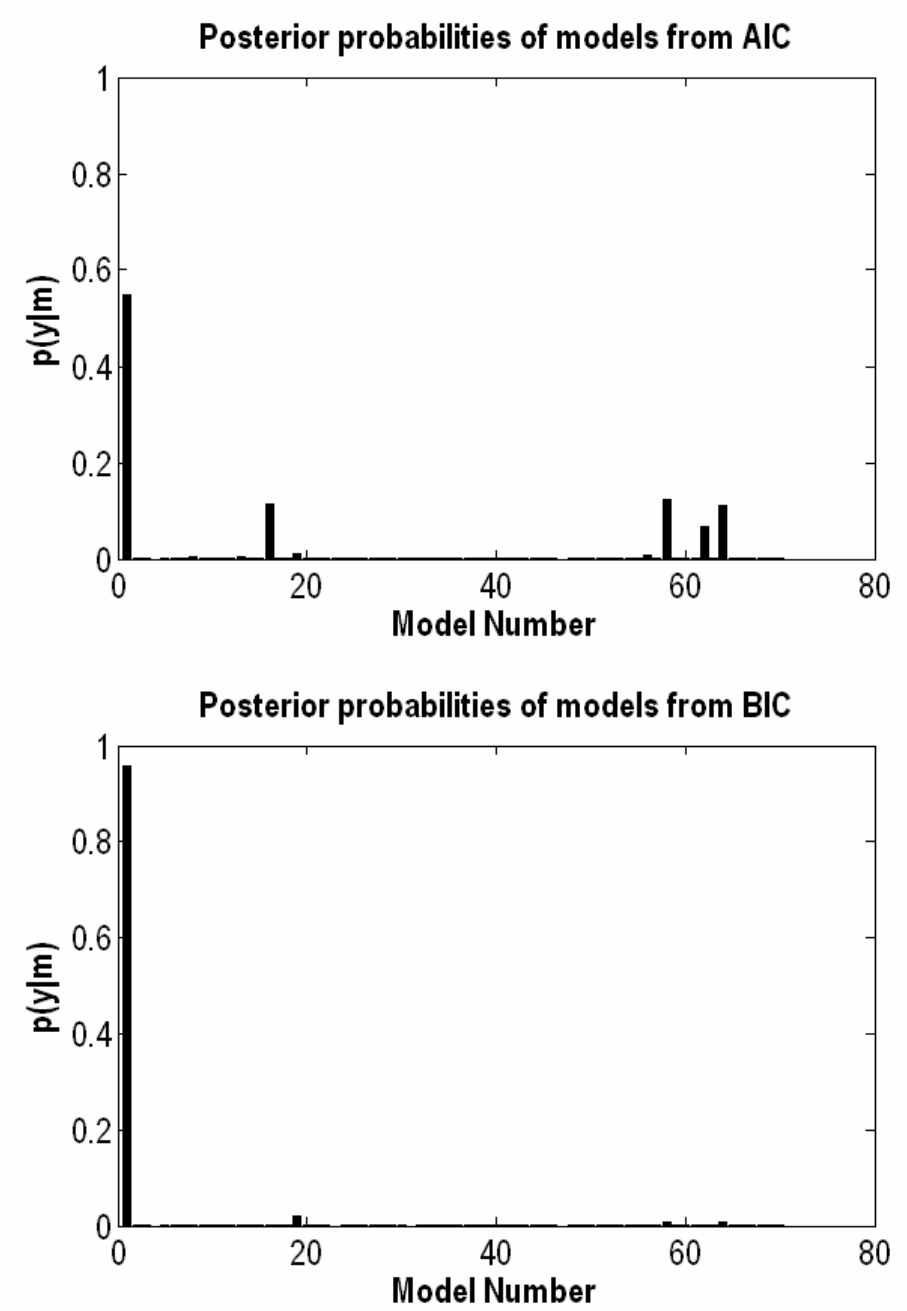

Subject 2

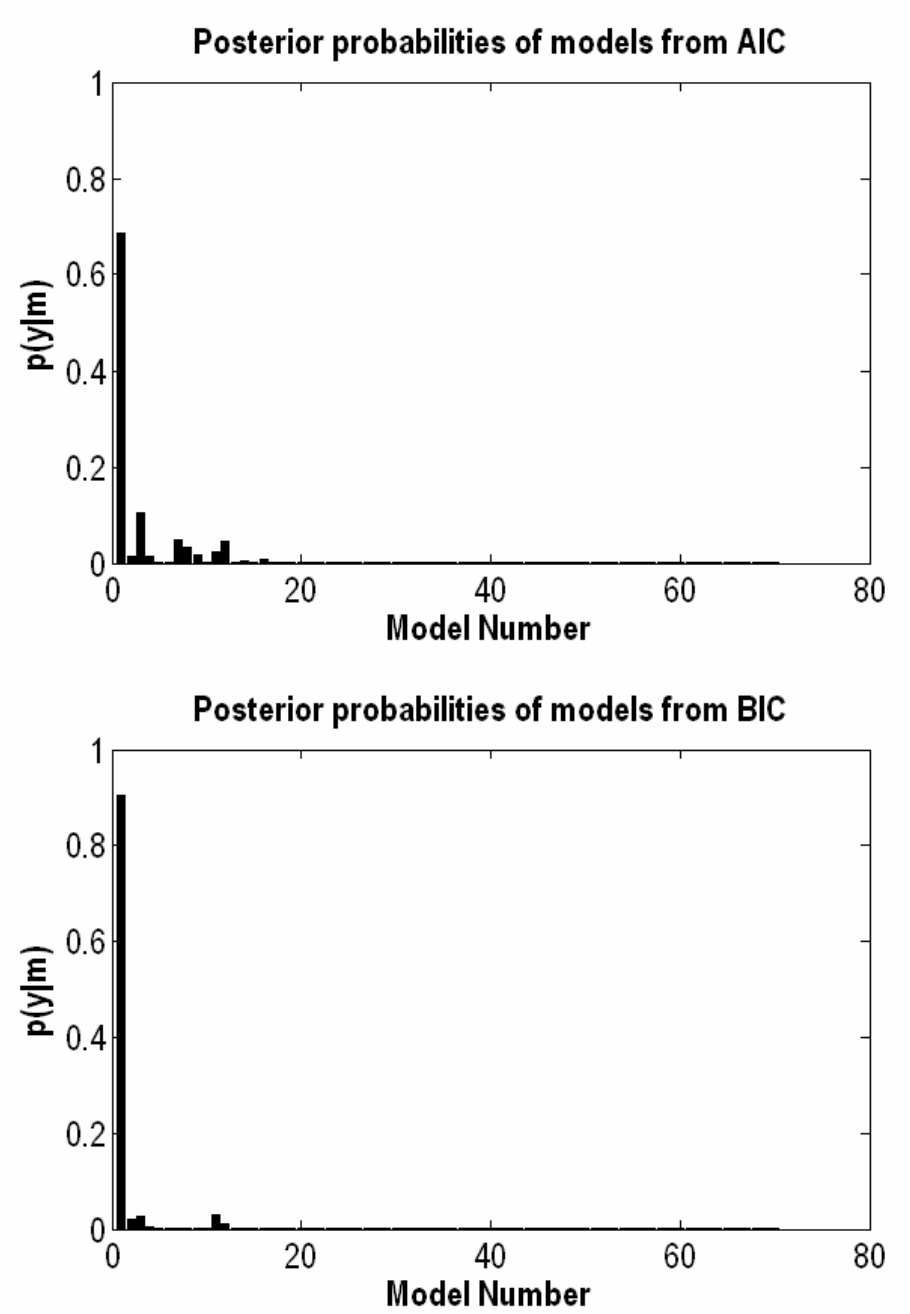

Subject 3

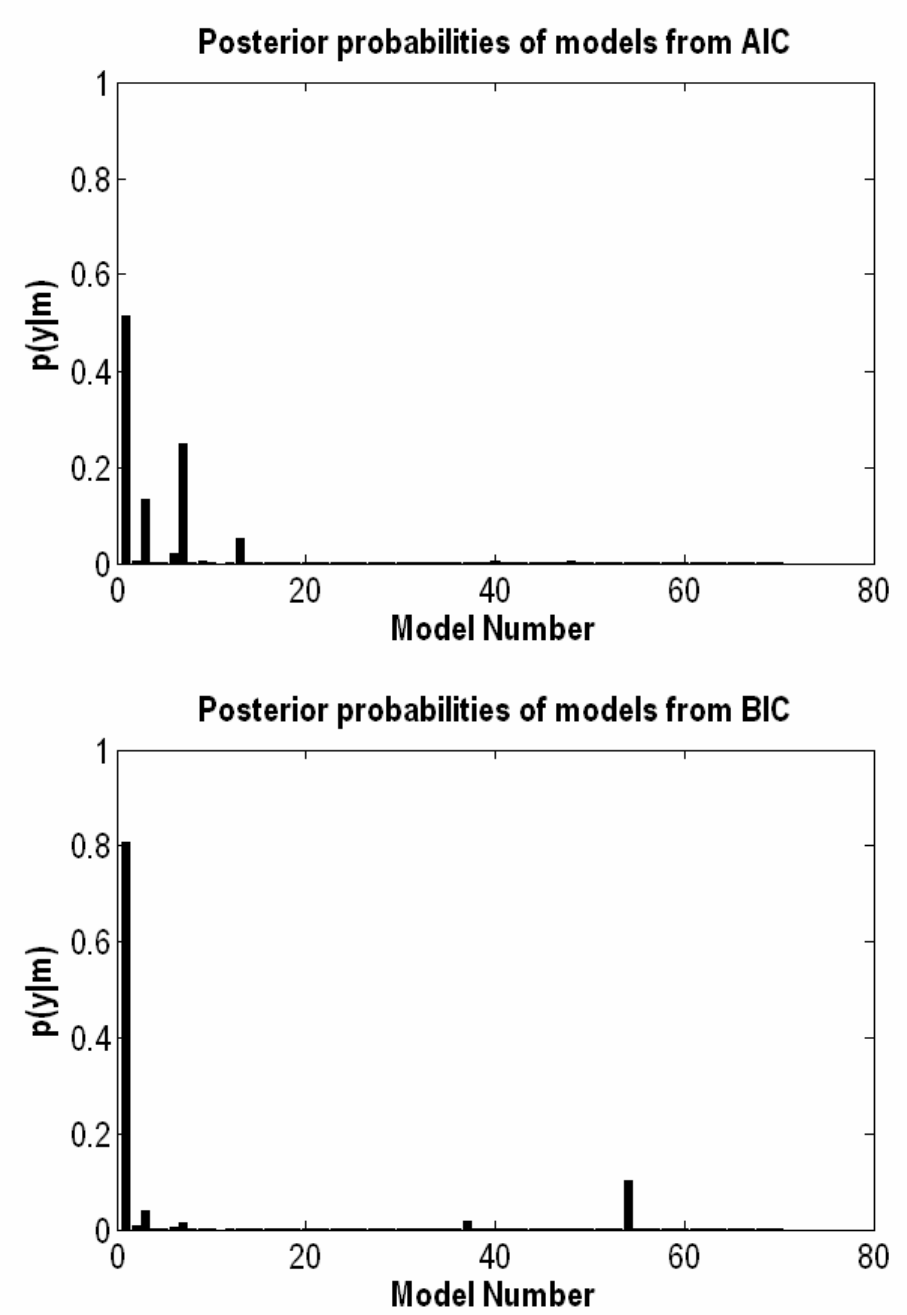

Subject 4

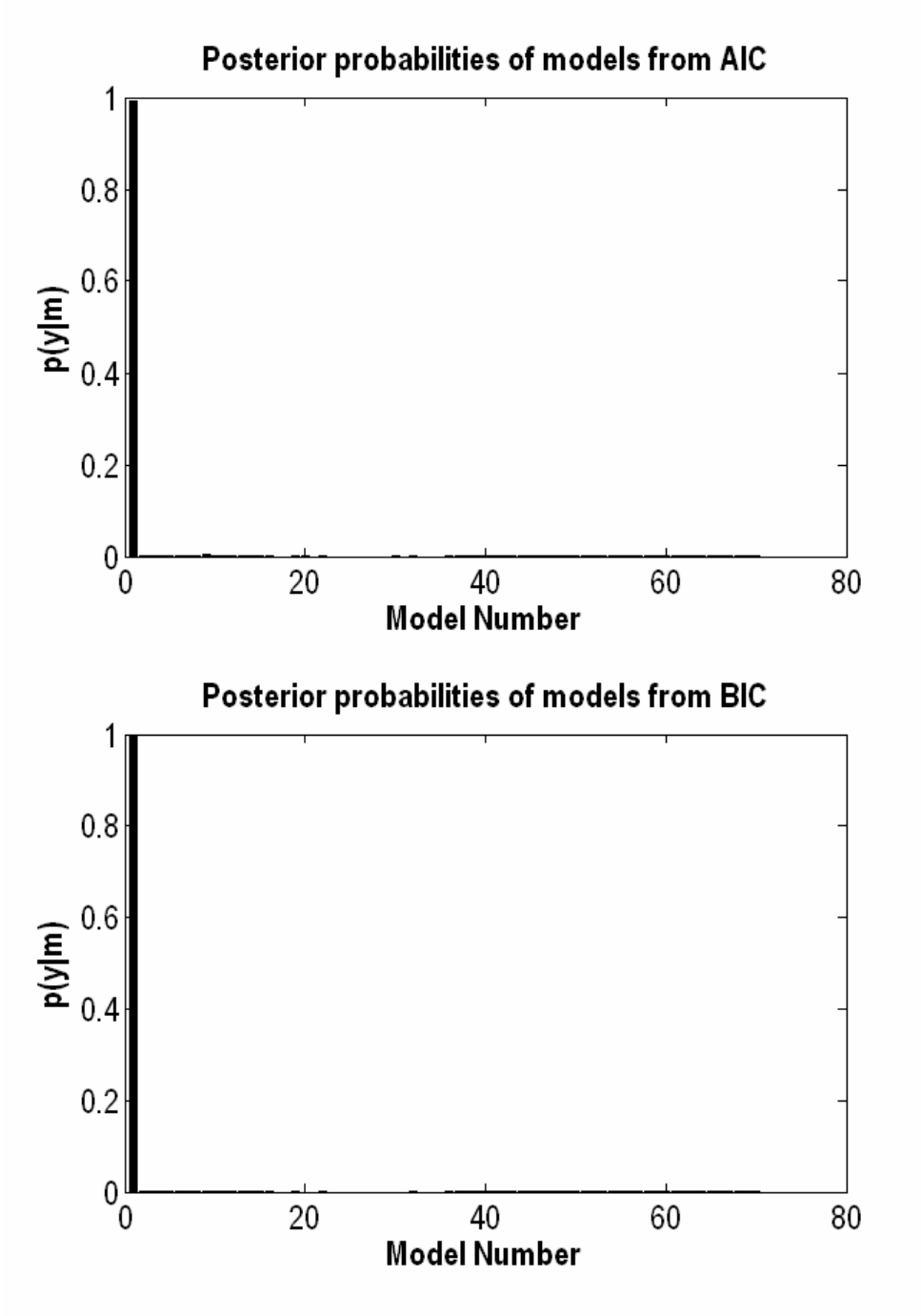

Subject 5

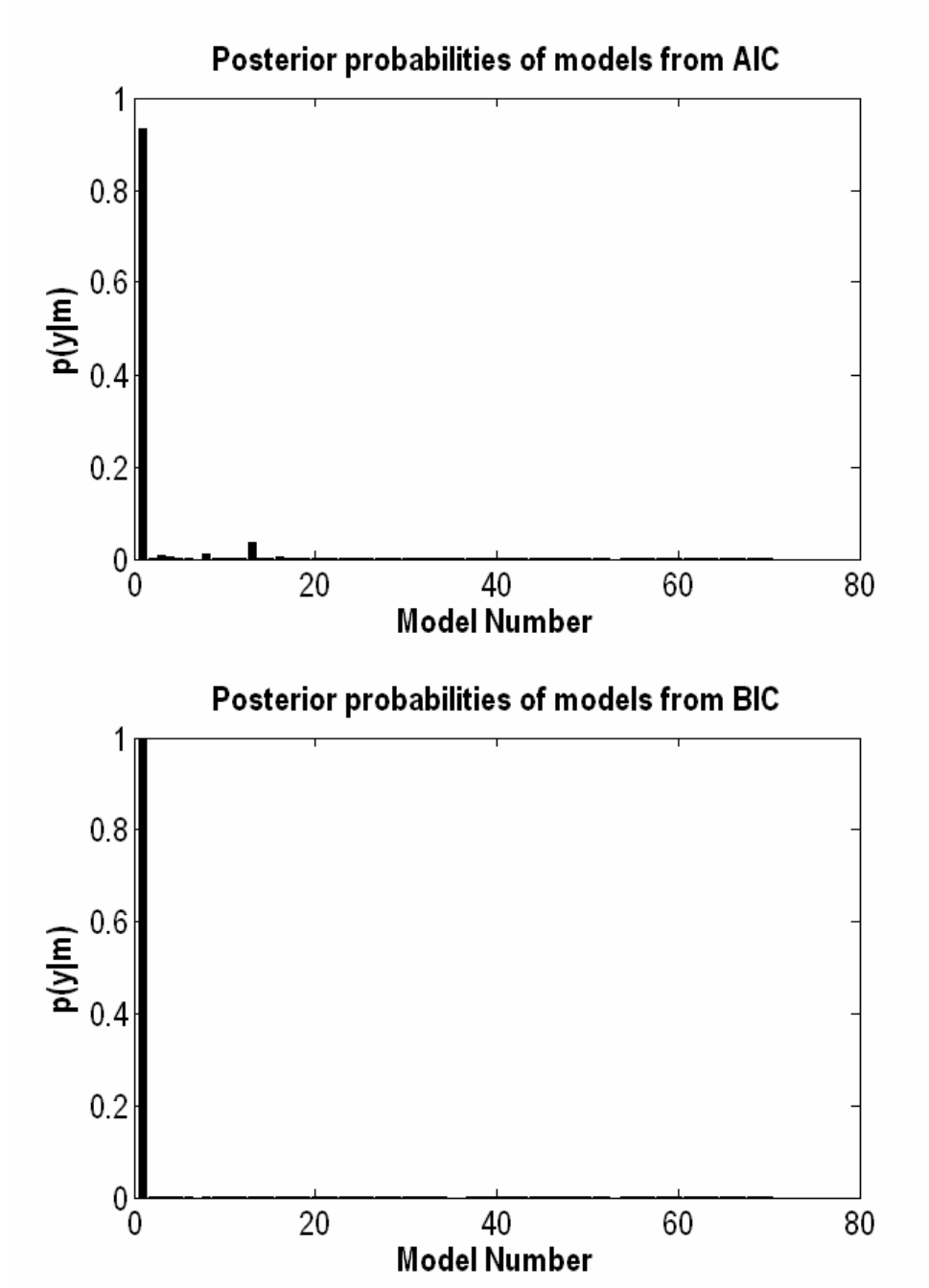

Subject 6

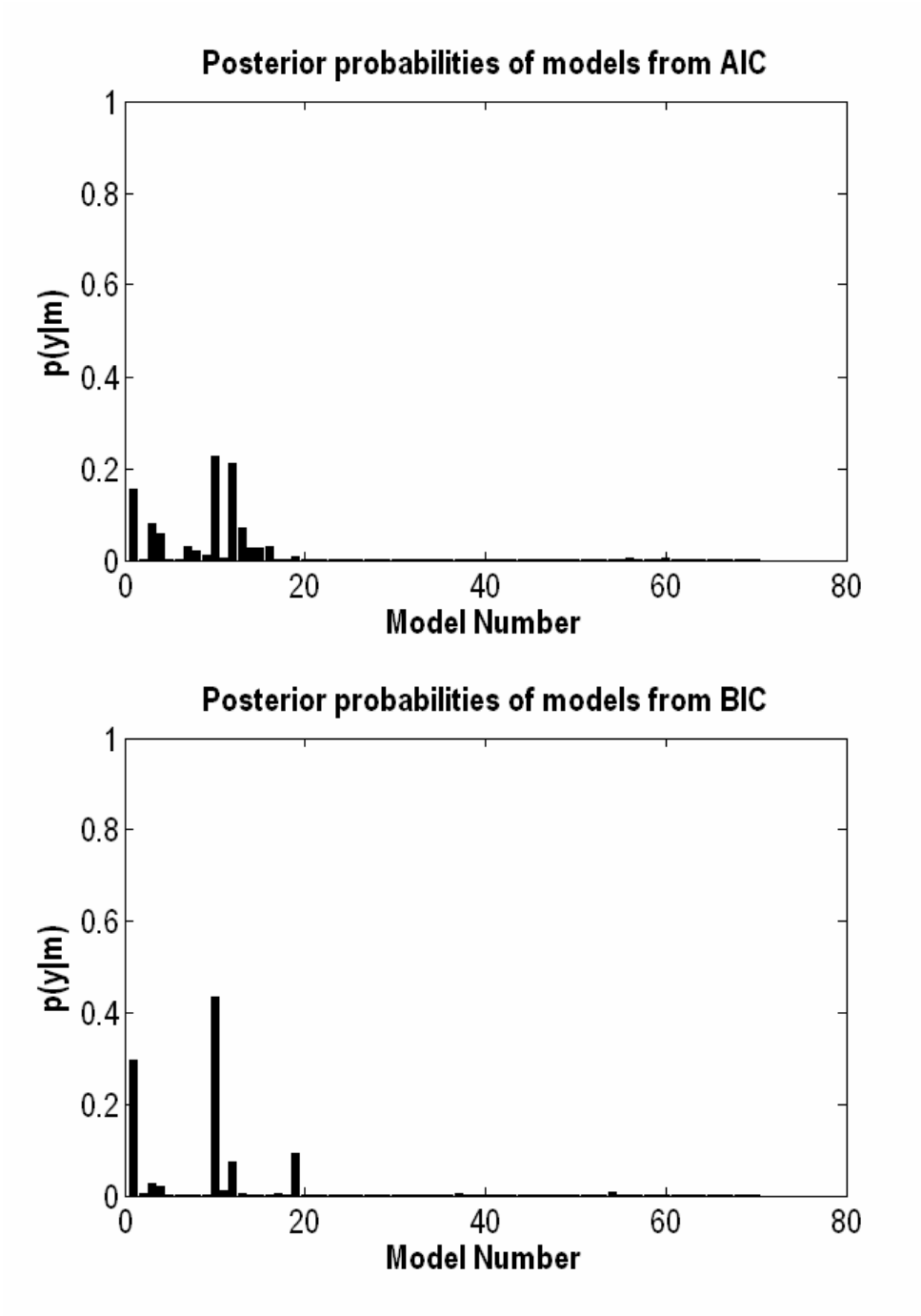

Subject 7

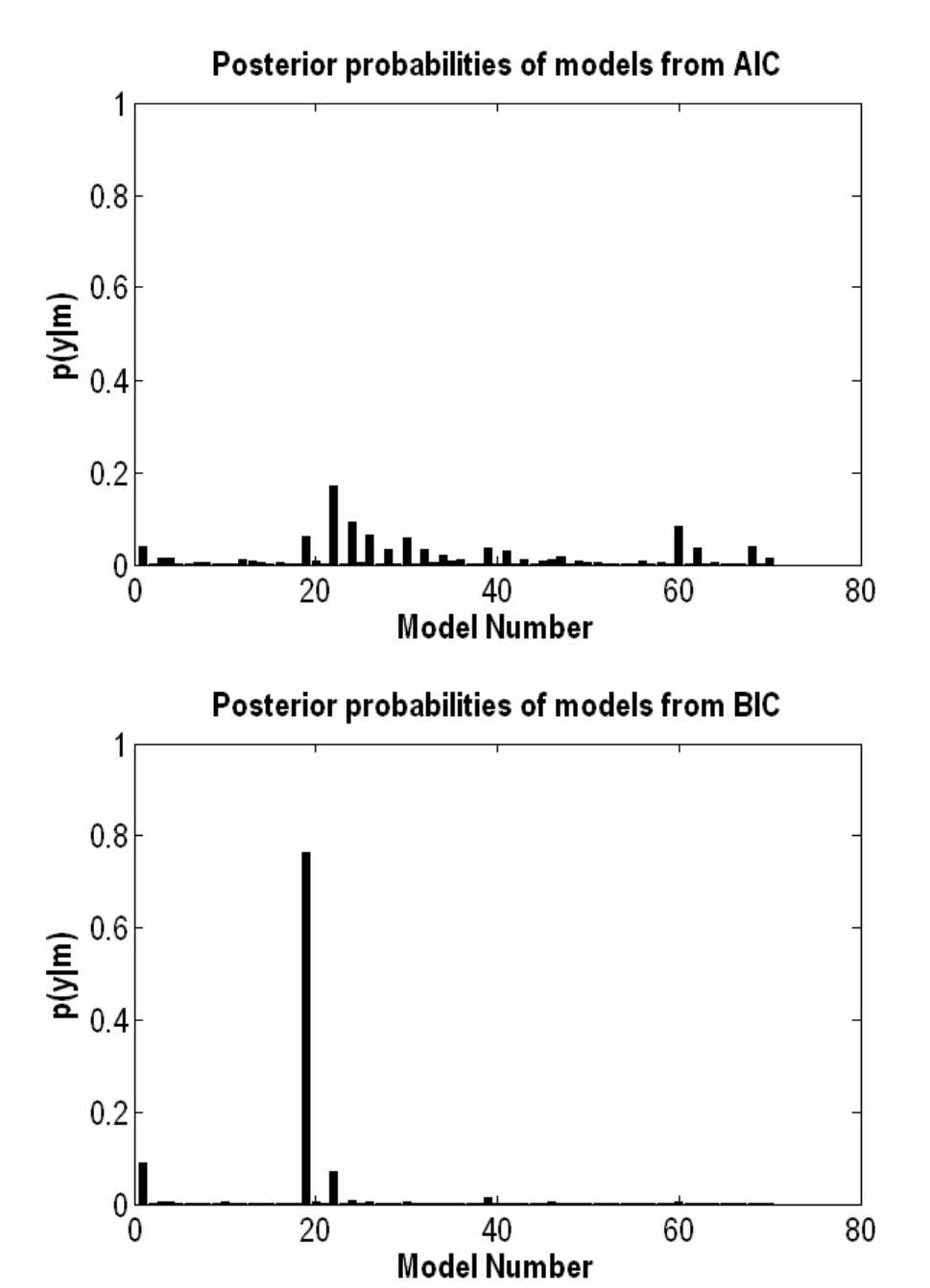

Subject 8
