# Supplementary material for: Hierarchical Processing of Auditory Objects in Humans
Source: PLoS Comput Biol. 2007 Jun 1;3(6):e100. doi: 10.1371/journal.pcbi.0030100 (PMC1885275; doi:10.1371/journal.pcbi.0030100)
Supplement: Supplementary file 3 [file pcbi.0030100.sg003.doc]

**(a)**

**(b)**

**(c)**

Figure S3: Time series plots of measured and predicted BOLD response from (a) HG (b) PT and (c) STS for subject 5
